# New Insights in the Pathogenesis of Atopic Disease

**Published:** 2009-04-25

**Authors:** Ionescu John G.

**Affiliations:** * Research Department of the Spezialklinik Neukirchen, Germany

**Keywords:** Pollutants, Immunotoxic / neurotoxic action, Allergy, Atopic eczema, Pseudoallergic reactions, Intestinal microflora, Intestinal permeability, Amalgam fillings, Mercury

## Abstract

A causal link between the increasing environmental pollution and the fast spreading of allergic diseases is currently discussed.

The exogenic and endogenic noxious agents contributing to the total environmental load are primarily acting through immunotoxic, sensitizing and neurotoxic mechanisms in animal experiments and in humans. Beside classic allergic-triggering factors (allergen potency, intermittent exposure to different allergen concentrations, presence of microbial bodies and sensitizing phenols), the adjuvant role of environmental pollutants gains increasing importance in allergy induction.

Our therapy experience with more than 18.000 atopic eczema patients shows that beside allergic reactions pseudoallergic mechanisms through toxic environmental agents (formaldehyde, industrial and traffic smog, wood preservatives, microbial toxins, additive-rich food, nicotine, alcohol, pesticides, solvents, amalgam-heavy metals) are increasingly incriminated as causal factors for the complex symptomatology.

The avoidance and elimination of such triggering factors before and during pregnancy and in early childhood may result in a significant decrease of the incidence of atopic diseases.

Recent statistics of the Federal Republic of Germany report more than 25 million cases of allergies; nearly every week mass media report a new environmental problem, so that a relationship between environmental load and break-out of allergies becomes more and more apparent.

This development was nearly unknown until the 2nd half of the 20th century, but just in the last 20-30 years, parallel to the increasing environmental pollution, a rapid spreading of allergies was noticed, especially in major cities.

**Pollutants responsible for total environmental load**

We may actually count more than 7×10^6 chemical compounds with a yearly increasing rate of more than 250 000 new substances. More than 50,000 are used daily. They contribute to the environmental load of the air by exhaust fumes (SO2, NO2, CO) of cars, industrial factories and power stations, industrial dust, excess of ozone as well as the chemical burden of soil and water with pesticides, fertilizers, insecticides, heavy metals and chemical or radioactive residues. Virtually no one can avoid over longer time the negative effects of different toxins like asbestos, formaldehyde, wood preservatives, adhesives, oils, petrol and solvents, resins, detergents and heavy metals. Besides, daily intake of fast food products may cause various side effects through preservatives, dye-stuffs, binding substances, gelatines, emulsifiers and taste intensifiers giving our food a long stability, an appetizing appearance and the desired taste. Cosmetic ingredients, drugs and pharmaceutical products as well as dying-stuff and synthetic materials of the textile industry are also potential triggers of intolerance reactions.

Furthermore, allergic and chemical sensitive patients frequently react upon exposure to electromagnetic fields of different electrical equipments like PC's, TV, HiFi- and other house devices, antennas and power lines as well as to radioactive residues from environmental and medical sources. The burden of the daily psycho-social stress (distress) added to the above factors, makes a first sum-up of the body’s load through **exogenous environmental challenge**.

The function of different body systems can also be impaired when chronic infections through bacteria and yeasts are colonizing the mucosa of the respiratory, urinary and intestinal tract. Their metabolic and catabolic products (endo-, exo- and mycotoxins, indol, scatol, phenol, biogenic amines etc.) together with the mobilization of toxic heavy metals from pessars, amalgam fillings, dental crowns and other prostheses, ionogens of different implantations (synthetic material, silicon, acrylate, dental cement) are making a second group of **endogenous factors** also contributing to a toxic cumulative raise of the oxidative stress. American authors introduced some years ago the term "Total Environmental Load" as standard for the body's total burden through pollutants [**1**].

The contact with the described noxious agents happens primarily in the respiratory system through inhalated stuff, in the intestinal tract through food and water as well as through the skin and causes different effects. Via skin and mucosa most toxins disturb the metabolic processes and cellular structures of the immune system and / or of the central nervous system. As a direct consequence, immunotoxic or mitogenic effects of the blood cell subpopulations [**2**,**3**] are observed as well as the decrease of the secretory immune globulins [**4**,**5**], normally connected with an increased susceptibility for infections of the skin, mucosa and intestine, as often found in allergic patients [**6**].

The various neurotoxic effects of pollutants can break out as headache, dizziness, concentration problems, tremble, insomnia and disturbances of the cardiac rhythm up to depressions and partial paralysis [**7**-**10**]. A disturbed release of catecholamines in chemical sensitive hyperkinetic (dopamine) and atopic (noradrenalin) patients was also reported [**11**, **12**]. A part of the pollutants is stored in fat tissue, bones and nervous system and will occasionally be mobilized with negative effects for the patients. This was clearly documented by analytic investigations of the fat tissue (**Table 1**).

**Table 1:** Levels of different organo chlorinated compounds in human fatty tissue [80]

	Year	Number of tests	Average levels (mg/kg) of					
			p,p'- DDE	pp-DDT	Dieldein	HCB	total HCH	PCB
Belgium	1975	60	6.5	1.52	0.26	1.36	0.76*	0.91
Kanada	1972	168	2.095	0.439	0.069	0.06	0.065	0.91
Germany (West)		282	4.4	1.1	0.14	5.6	0.99	8.3
Greece		50	7.86	1.99	0.23	3.84	0.98**	
Japan	1974	30	2.91	0.68			2.36	1.04
Northern Ireland	1975	11	1.60	0.34	0.10	0.15	0.45	
Spain	1977	40	2.268	1.781	0.150		0.062	
Switzerland	1971/72	12	3.8	1.6	0.29	1.9	0.90	1.0
England	1976/77	236	5.1	0.21	0.11	0.19	0.33	0.7
USA	1973/74	898	2.1		0.15		0.21*	
New Zealand	1973	51	4.4	0.46	0.21	0.31	0.49	0.82
Danmark	1972/73	78	3.7	0.6	0.12			3.8
* Measured as ß-HCH; ** measured as Lindan								

As such cases occurred more and more frequently, new medical notions like "Multiple Chemical Sensitivity" and "Sick Building Syndrome" associated with complex systemic symptoms were introduced in the last years. A part of the patients believes to have an allergy to environmental chemicals although this allergy is not evident by classic immunological tests. On the other side, allergists have noticed that most of their patients with asthma, atopic eczema, allergic rhinitis or urticaria are simultaneously environmental reactions showing an increased sensitivity to smallest concentrations of different pollutants and biogenic toxins. Their symptoms are mostly due to a mixture of allergic and pseudo allergic reactions. Experts already talk about **allergotoxicology** as a new interdisciplinary field including allergic and chemo-sensitive patients.

**Triggering factors of allergic reactions**

A complex relationship between different blood cells like macrophages, lymphocytes, eosinophiles, basophiles, monocytes and granulocytes, mediated by specific cytokines is known to play an important role in inducing a normal immune response or an allergic reaction with inflammatory components, respectively.

According to Gell and Coombs immediate and delayed allergic reactions of Type I to Type IV can happen, always with participation of the immune system. Besides a hereditary disposition for atopy being most intense if both parents are atopics (prevalence of the atopy of 60-80% in the filial generation), several trigger factors may play an important role for the induction of an allergy. To these belong:

1. *Allergen potency and exposure*. An early stop of the breast feeding and the replacement with food formula based on strong allergens like cow milk, soy or food with parts of eggs and yeast are known as factors for an allergy induction in babies [**13**]. The chemical combination of the so-called haptens is hereby crucial for the allergen potency and the repeated intermittent exposition to different allergen concentrations is also a known prerequisite for the induction of allergy.

2. Microbial bodies like *Mycobacterium tuberculosis* or *Bordetella pertussis* [**14**] and different irritating substances like phenols (Freund’s adjuvant) can also mediate the induction of an IgE-response and allergy in animal experiments. A similar situation may happen in babies being early infected by facultative or obligate pathogen germs of the mother’s birth tract or in the hospital, leading to an early contamination of the intestine with an abnormal flora. Similar microbial conditions combined with production of large amounts of sensitizing decomposition products (e.g. biogenic amines, phenols) can also arise later from antibiotic treatments damaging the intestinal flora or under the influence of radiation, cytostatic or cortisone therapies, all with immunosuppressive effects.

Such dysbiotic conditions of the intestine are markers of allergic diseases [**15**]. They are reversing the normal priming of immunocompetent lymphocytes in the gut associated lymphoid tissue (GALT) towards increased numbers of Th2 cells, IL-4 generation and IgE-synthesis [**62**,**63**].

3. Different pollutants have an adjuvant role for the induction of allergies. Several mechanisms are possible:

- Damage of the skin and mucosal barriers through chemical, physical or microbial influences via direct injury of cell membranes and release of inflammatory agents like histamine, prostaglandins and leucotrienes, especially after pesticide exposure [**16**,**17**], alcohol and microbial toxins. The raised permeability of the mucosal barriers leads to an increased intake of allergens followed by sensitization [**18**].

- A raised IgE-production and outbreak of allergic symptoms after diesel exhaust particles [**19**], tobacco smoke [**20**,**21**], mercury compounds [**22**] and platinum salts [**23**] was also reported. The increased toxin concentrations can spread their effects on different cellular levels, depending on their absorption and irritation potential.

- Induction of the IgE-synthesis after toxin (e.g. formaldehyde) binding on serum proteins [**24**] with formation of new antigen structures.

- Conformational changes of the cellular surface after contact with heavy metals combined with sensitizing effects for antigen specific T-lymphocytes and leading to their proliferation and differentiation (contact allergies) [**25**,**26**].

- Intervention in the intermediary metabolism by influencing the structure and biological activity of different enzymatic systems, RNA, DNA and protein syntheses (methyl Hg inactivation of SH-proteins, DNase, ATPase and oxidative phosphorylation, alcohol and nicotine inhibition of MAO/DAO activities etc.) [**27**-**31**].

As a rule, symptoms of allergic and/or chemical sensitive patients are the consequence of interactions between their immune/detoxification systems and the influences of inappropriate food, exogenic pollutants and endogenous toxins.

This explains the polymorbid clinical status of such patients. The importance of preventive medical steps is evident - by avoiding relevant allergens and pollutants – and of an appropriate healthy life-style in the psychosocial respect as well.

**Allergotoxic factors in atopic eczema (AE)**

Our experience in the treatment of more than 18,000 atopic eczema, urticaria and contact dermatitis patients shows, that besides the allergic mechanisms more and more pseudo allergic reactions caused by toxic-irritative pollutants (formaldehyde, exhaust particles, additive-rich food, nicotine, wood preservatives, pesticides, heavy metals) are responsible for the complex symptoms. Intrauterine and postnatal influences of such factors were also reported [**32**,**33**].

Highly interesting in atopic eczema (AE) patients is the occurrence of allergic and pseudoallergic reactions against food and food additives, investigated in controlled studies before and after challenge meals [**18**]. Besides a distinct increase in the serum levels of circulating immune complexes and specific IgE and IgG4 antibodies against food, the investigation of the serum histamine levels before and 1/2 hour after challenge meals shows in AE patients a highly significant increase of the mediator after the food intake [**Fig. 1**].

**Fig. 1:**
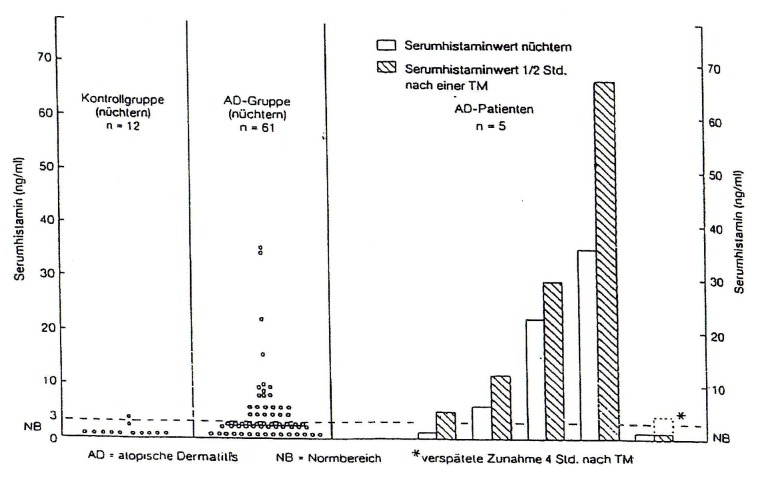
Serum histamine levels in atopic eczema patients

However, as the fasted serum of AE patients also contains high histamine levels, we investigated the catabolism of this biogenic amine.

Diaminoxidase (DAO) is the main splitting enzyme for histamine, but also monoaminoxidase (MAO) plays a role in its catabolic pathway. In a study about the MAO- and DAO-activity in thrombocyte-rich plasma of AE patients and healthy controls we were able to demonstrate that the activities of MAO and DAO were significantly decreased in AE patients when compared to controls. Simultaneously we noticed significantly increased histamine levels in the same fasted AE patients (**Table 2**) [**64**]. High concentrations of biogenic amines, e.g. putrescine, octopamine and histamine, are known inhibitors of these catabolic enzymes. Further inhibitory factors are food additives, heavy metals, alcohol and nicotine, whereby alcohol additionally leads to an increased absorption of biogenic amines from the intestinal tract. Some drugs, for example certain antidepressants, act as MAO blocker as well. On the other side, the cofactors for MAO (iron and FAD) are decreased in AE patients, the cofactors for DAO (copper and pyridoxalphosphate) are nearly normal, suggesting excesses of the enzyme inhibitors mentioned above [**31**].

These findings explain the pseudoallergic, non-immunological mediated reactions against certain foods like frozen fish containing 10 times higher histamine levels than fresh fish. In different varieties of blue cheese, sour cabbage, pickled cucumbers or ketchup high concentrations of histamine can be found, too. Pseudoallergic reactions against other biogenic amines like tyramine, octopamine, phenyl ethylamine or putrescine may also happen because splitting enzymes like MAO and DAO are inhibited or lacking. High concentrations of the biogenic amin tyramin are found for instance in sausages, beer, red wine, champagne and certain varieties of cheese. 

**Table 2:** Monoamine and diamine oxidase activities in platelet-rich plasma of atopic eczema patients and healthy controls

	Type B monoamine oxidase (mmol min -1 l -1)	Diamine oxidase (mmol min -1 l -1)	Histamine (ng ml -1)
Atopic eczema patients	0.223 ± 0.11 (n = 19)	0.270 ± 0.089 (n = 18)	6.63 ± 1.64 (n = 19)
Controls	0.371 ± 0.085 (n = 11)	0.511 ± 0.125 (n = 10)	2.15 ± 0.96 (n = 10)
Significance Student t test	P < 0.05	P < 0.001	P < 0.0001

The increased intestinal permeability in atopic patients enables a significantly raised antigen absorption which in turn leads to higher levels of circulating immune complexes, activation of the complement system and of the coagulation cascade and to the IgE mediated degranulation of the granulocytes and basophiles [**18**]. However, a direct correlation between the increased intestinal permeability on the one side and the serum histamine levels or the presence of IgE-containing CIC's on the other side was not found.

The reason for the increased intestinal permeability [**Fig. 2**] lies in the significant intestinal dysbiosis of most AE patients depicting an excess of facultative pathogenic bacteria or yeasts and a decrease of the beneficial, lactic acid producing flora, respectively [**15**].

In a previous study with 110 AE patients we found that in nearly 85% of the investigated cases exist the dramatically lowered levels of lactobacilli and bifid bacteria was paralleled by a strong increase of facultative pathogenic germs like haemolytic *E. coli*, *Klebsiella sp.*, *Proteus sp.*, *Clostridium sp.* and fungi like *Candida* or *Geotrichum sp.* (**Table 3**).

**Fig. 2:**
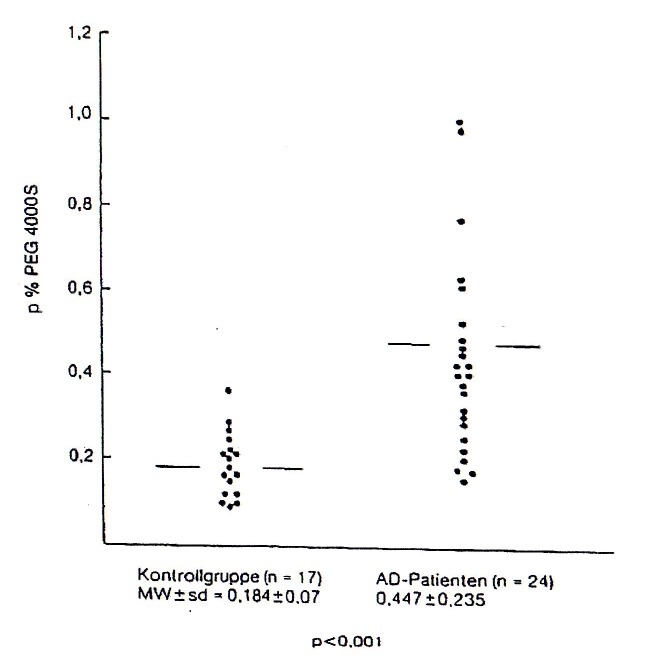
Intestinal permeability in atopic eczema patients, measured by procentual determination of the Urinal PEG 4000 S after 24 hrs

**Table 3:** Intestinal microflora of 110 atopic eczema patients and 30 healthy controls

	Lactobacilli	Bifidobacteria	haemolyt. coliforms	Klebsiella	Proteus	pathogenic Clostridia	Candida / Geotrichum
Normal range (germs / g moist stool)	> 10^6	> 10^8	< 10^4	< 10^4	< 10^4	< 10^5	< 10^3
AE-patients n = 110 (p%)	absent or < 10^4 76 (69%)	< 10^7 31 (28.2 %)	> 10^6 52 (47.3%)	> 10^6 36 (32.7 %)	> 10^5 22 (20%)	> 10^6 40 (36.3%)	10^4-10^7 48 (43.6%)
controls n = 30 (p%)	2 x 10^4 3 (10%)	> 10^8 30 (100%)	3 x 10^5 2 (6.6%)	< 10^4 30 (100%)	< 10^4 30 (100%)	< 10^5 30 (100%)	2.5 x 10^4 3 (10 %)

A lactose malabsorption test performed during the same study demonstrated significantly reduced levels of galactose in blood and urine of the AE patients, when compared to healthy controls. It is well known that the existing lactose malabsorption originates in a deficiency of lactase activity. The frequent intolerance reactions against sugar and sugar products in atopic patients are pseudoallergic reactions caused by a secondary disaccharides deficiency inducing diarrhoea, intestinal colics, migraine, skin rushes and oedema. The non-digested sugars in the gut support a strong increase of pathogenic bacteria and above all of fungi. *Candida albicans* and haemolytic *E. coli* are the main inhibitors of the lactase activity [**34**,**35**].

By fermentation, different intestinal yeast and bacterial strains transform the ingested carbohydrates in organic alcohols [**36**] and short chain fatty acids with narcotic effects [**37**, **38**] explaining the significant postprandial tiredness and alcohol intolerance of the patients, as well as their increased intestinal permeability. Most *Candida* strains generate phospholipase A2, whereby membrane phospholipids can be splited to archidonic acid and then oxidized to prostaglandins and leucotrienes with an important inflammatory potential. Toxic decomposition products of meat (indol, scatol, phenols, biogenic amines) produced by a putrefactive flora (*Clostridium, Bacteroides sp.*, pathogenic *Enterobacteriacea*e) are also increasing the irritability of the skin, mucosa and nervous system and act as adjuvant factors (e.g. phenols) for the induction of the IgE synthesis (Freund’s adjuvant).

Therefore the identification and elimination of chronic microbial foci of the skin, lungs and especially of the gut is essential for a successful therapy in atopic patients. 

## Disturbed energy metabolism and regulation factors

Further side-effects of a disturbed intestinal flora were discussed in detail in a former study [**15**]. The reduced absorption of carbohydrates caused by inhibition of dissacharidases together with a disturbance of the intermediary metabolism by environmental toxins of different kind (PCP, pesticides, industrial smog, heavy metals, polychlorinated biphenyls, microbial poisons etc.) produces a more and more reduced formation of energy-rich substances (ATP) in AE patients shown in several investigations [*Ionescu G, Benkert P*, 1988]. This is one of the main important causes for low levels of cyclic nucleotides (cAMP) with regulation/ control function when releasing inhibition mediators. The cAMP-levels are additionally reduced by simultaneous blockage of beta-adrenoreceptors [**39**,**40**] and by the increased activity of the camp splitting enzyme phosphodiesterase [**41**].

## Heavy metal burden in atopic patients

In the last years it was more and more reported about the immune- and allergotoxic relevance of heavy metal burden (cadmium, lead, platinum, copper) [**2**,**42**-**45**].

Mercury plays a specific role, even because of the intensive discussion about "amalgam". It is important to divide between the allergic potential of mercury (up to 17% allergy incidence with positive epicutan testing’s) and his chronic-toxic potential leading especially in allergic patients to an accelerated summation effect with other pollutants. 

Some examples of several mechanisms for the attack are referred:

– Binding to serum and cell proteins (albumin, coenzyme A, SH-proteins) with interactions in the intermediary metabolism [**27**].

– Rise of the cellular mitosis rate in lymphocytic populations [**46**].

– Induction of the IgE-synthesis with increase of the allergen specific IgE-answer in rats [**22**].

– Decrease of total T-lymphocytes, T-helper cells and natural killer cells after mercury release from amalgam fillings [**47**,**48**].

– Stimulation of inflammatory reactions by activation of the corresponding enzymatic systems (for example collagenases [**49**]).

– Exceptionally important for patients is the transformation of the ionisized Hg2+-form through methylation in a more toxic, lipid soluble organic compound (methyl mercury) [**27**]. Oral and intestinal bacteria (streptococci, clostridia) [**50**] and especially yeasts (*Candida albicans*) [**51**] accomplish this process and partly explain the tolerance differences between amalgam carriers.

Although the clinical relevance of these results for the pathogenesis of atopic eczema is still controversial, the most important mercury sources remain always the same: as addition in different drugs (liniments, drops, and vaccines), fish-rich food, entrails (0.3-2.3µg/day) and especially amalgam fillings (3-17µg mercury vapour/day [**27**]).

Mercury possesses an evident affinity for the epithelial cells of the gastrointestinal tract and the skin, for hair, thyroid gland, liver, pancreas, kidneys and the brain (above all the grey substance as well as in central areas of the brain stem and the cerebral cortex) [**52**-**54**]. Spin-tomographical investigations of the head are not practicable, if patients carry dental alloys in the stoma [**55**].

Our own investigations in atopic eczema, urticaria and psoriasis patients show by means of the so-called "chewing-gum test" a considerable metal release, directly correlating to the number of fillings [**56**]. This phenomenon is not only significant for the patients, but also for pregnant women and mothers, then according to newer findings the release of mercury leads to a transplacental burden of the foetal tissues [**32**,**33**] as well as of the babies by mother's milk [**57**]. The release from the fillings is more severe after intake of hot drinks (coffee, tea), fruit juices, and fresh fruits or during gum chewing. The allergotoxic effect of mercury is often multiplied through synergistic effects of other pollutants (pesticides, dioxins, furans etc.) from breast-milk.

According to our experience with over 18,000 allergic skin patients two facts are certain:

a) Dentists drill work in the amalgam fillings of allergic patients without appropriate protection (cofferdam, antioxidants) generally lead to a rapid worsening of the symptoms, and

b) Therapy resistant, recurrent atopic eczema of the head, neck and face skin as well as chronic rhinitis and pharyngitis greatly improve or subsides after the removal of amalgam fillings followed by adequate mobilisation procedures [**58**].

## Prophylaxis and therapy

Important preventive steps for allergic patients may already be accomplished in pregnant women, whereby the avoidance of strong allergens in food, alcohol, nicotine and exogenic toxins can lead to a decrease of the baby's atopic risk [**13**]. Also the clearing of endogenic load factors like chronic infections of the skin, mucosa, birth tract and intestine find its justified use.

An allergen-poor diet offered in a rotation system, together with the clearing of microbial foci, toxin mobilisations, immunmodulation and psychological care are important promoters of a long-term recovery [**59**,**60**].

Main therapy steps in atopic patients may therefore include: 

- Immediate compensation of the redox and free radical status of the blood.

- Elimination of pathogenic germs of the skin, mucosa, respiratory system, urinary tract and intestine.

- Restoration of a healthy intestinal flora and function, by means of antimycotics, plant extracts, appropriate food and lactic acid producing bacteria.

- Reduction of increased levels of pathogenic substances in blood as biogenic amines, endotoxines, pathogenic antibodies and circulating immune complexes.

- Specific or unspecific modulation of the humoral and cellular immune function by intake of immunoglobulines, selected thymus extracts and other biological agents.

- Modulation of neurohormonal imbalance (noradrenalin, dopamine, serotonin).

- Activation of the detox systems of the liver and blood (alcohol/aldehyde dehydrogenase, phenoloxidase, monoaminoxidase, methyltransferase, GST).

- Compensation of deficits of vitamins, minerals, trace elements, amino acids, enzymes, essential fatty acids and energetic substances by appropriate substitution therapy.

- Preparation of appropriate diet plans considering the individual allergic and pseudoallergic intolerance reactions.

- Dietary treatment: Based on the individual spectrum of food intolerances, there is no ideal diet plan for all atopic patients. An extensive panel of diagnostic procedures for allergic and pseudoallergic reactions against food antigens and additives is therefore imperative before completing a diet plan. These include detection of food allergens by means of specific IgE and IgG4 antibodies, histamine and free radical release before and after challenge with foods or additives, identification of lactose, fructose or sucrose intolerances as well as pseudoallergic reactions resulting from microbial fermentation/putrefaction processes in the gut.

## Conclusions

When we investigate the complexity of the atopic disease than it is easily comprehensible that a symptomatic therapy with cortisone, radiation or antihistamines gives hardly a chance for a long term recovery.

On the contrary, a functional concept should include a targeted diagnosis of individual deviations in the intermediary metabolism, immunological state and nutrition status to offer the appropriate therapeutically steps. The long lasting recovery achieved in more than 80% of all cases justifies this integrative therapy concept and won the acceptance of physicians, patient organisations and all health insurances who integrated the clinic in the official German health system.
